# A node influence ranking algorithm combining k-shell iteration and node degree

**DOI:** 10.1371/journal.pone.0328381

**Published:** 2025-07-24

**Authors:** Yating Ji, Lequn Liu, Shujia Li, Pu Lu, Qimei Tang

**Affiliations:** 1 Information Management Office, Hefei Normal University, Hefei, China; 2 Network and Information Management Office, Anhui Medical University, Hefei, China; 3 Key Laboratory of Philosophy and Social Science of Anhui Province on Adolescent Mental, Health and Crisis Intelligence Intervention, Hefei Normal University, Hefei, China; Asansol Polytechnic, INDIA

## Abstract

Identifying key nodes in complex networks holds significant application value in fields such as information dissemination and disease spread. The traditional K-shell decomposition method has low time complexity and is suitable for large-scale complex networks; however, it only considers global positional information, leading to lower discrimination. To improve the K-shell decomposition method, many approaches have been proposed by researchers. However, there no algorithm has yet that simultaneously uses the iteration factor and degree to further distinguish nodes with the same K-shell value. To address this issue, we propose a node influence ranking algorithm that integrates K-shell iteration, node degree, and neighbor information, considering both global network position and local topology. Through simulation experiments on eight networks, it was verified that this method provides more accurate ranking results compared to dc, bc, cc, k-shell, Ks + , KSIF, LGI and DCK methods on eight networks, with an average accuracy improvement of 5.15% over the second-best algorithm. In identifying the top 10 key nodes, the KTD algorithm demonstrates higher accuracy than other methods. Additionally, it shows high discriminative power and good time performance, making it suitable for large-scale complex networks.

## Introduction

Many real-world domains, such as infrastructure networks, transportation hubs, information dissemination, and disease spread, can be modeled as complex networks, which are network structures composed of numerous nodes and relationships between nodes [1]. There are important nodes in these complex networks, which influence the structural characteristics of the whole network and the speed of information dissemination [2]. Identifying these influential nodes has many practical applications, such as ensuring the stability of key nodes in power networks to prevent widespread outages [3–4], using influential users in public opinion events to control sentiment [5], and pinpointing critical nodes in disease networks to manage disease spread [6–7]. Therefore, research on node influence ranking algorithms holds significant theoretical value and practical importance.

Researchers have proposed various methods for ranking node influence in complex networks. The classic neighborhood-based algorithm is degree centrality (dc) [8], which measures node influence by the number of neighbors. Building on degree centrality, researchers have introduced methods such as semi-local centrality [9] and H-index centrality [10]. Semi-local centrality extends the neighborhood to the fourth degree, while H-index centrality takes into account the degree values of neighbors. Although neighborhood-based algorithms have low time complexity, they do not consider global characteristics like network position, resulting in lower accuracy [11]. Path-based classic algorithms include betweenness centrality (bc) [12] and closeness centrality (cc) [13], which measure node influence based on the number of shortest paths passing through the node and the average distance to all other nodes, respectively. While path-based algorithms account for global information, their high time complexity makes them unsuitable for large-scale complex networks [14]. Classic eigenvector-based algorithms include eigenvector centrality [15], PageRank [16], and LeaderRank [17]. These algorithms consider both the quantity and quality of neighboring nodes. However, in networks with high local clustering, some nodes may appear to have inflated influence, and these algorithms also have high time complexity, limiting their applicability to large-scale complex networks [18]. A classic global-position-based algorithm is the K-shell decomposition method (ks) [19], which layers nodes according to their network position, assuming that nodes closer to the network core have greater influence. While K-shell decomposition has low time complexity and is suitable for large-scale complex networks, its coarse granularity leads to many nodes with similar influence, resulting in lower precision [20].

To improve the accuracy of the K-shell decomposition method, researchers have proposed several enhancements. Zeng et al. (2013) introduced the Mixed Degree Decomposition (MDD) method [21], which simultaneously considers the influence of both remaining and removed neighbors on node influence. Bae et al. (2014) proposed the Extended K-shell method (ks+) [22], which integrates node degree and the maximum degree to extend the K-shell approach. Qiu et al. (2021) developed an algorithm for ranking node importance based on node degree, clustering coefficient, and network position (Local Influence and Global Influence, LGI) [23]. Wang et al. (2016) proposed the K-shell Iteration Factor (KS_IF) [24], which incorporates iterative information generated during K-shell decomposition. Zareie et al. (2018) proposed a Hierarchical Approach (HKS) that evaluates node influence by incorporating the b-index (distance from the periphery) and the f-index (proximity to the core), iteratively refining influence scores and computing the HKS index to achieve more accurate node ranking [25]. Many researchers have further incorporated iterative factors into the study of node influence ranking. Zareie et al. (2020) used iterative factors to hierarchically partition the network and considered the common hierarchy between a node and its neighborhood set (ECRM) [26]. They proposed a key node identification algorithm based on neighborhood correlation coefficients. Wang et al. (2024) combined iterative factors with information entropy to identify important nodes at each network level, from the periphery to the core (IE_+_) [27]. However, while these methods enhance the K-shell approach in various ways, they still have limitations in terms of capturing both local and global topological effects on node influence. For example, while KS+ and MDD improve the node ranking by considering node degree and local connectivity, they do not fully account for iterative effects during the decomposition process. Similarly, methods like LGI and KS_IF improve the ranking accuracy but do not explicitly combine global network position and local topology in a unified framework. ECRM relies on the level overlap between a node and its neighbors. When this overlap is weak, the influence estimation can become unreliable. IE + is sensitive to the distribution of network data when applying entropy-based weights. Uneven data distributions may distort weights and affect the identification of key nodes.

In addition, there are other related methods. Aman Ullah et al. (2021) proposed a Local-and-Global-Centrality (LGC) measuring algorithm, which identifies key nodes in complex networks by considering the node’s degree, a tunable parameter, and the shortest path between nodes [28]. This method relies on tunable parameters, which may be very sensitive to parameter selection, leading to performance fluctuations in dynamic networks. HamaKarim et al. (2023) developed a k-shell-based algorithm that identifies influential nodes by partitioning the network into communities, weighting edges, evaluating node spread power, and selecting the most impactful nodes for propagation [29]. The method evaluates node influence through community partitioning, but accurate community detection can be challenging in some networks, leading to inaccurate influence assessments. Zhao et al. (2023) developed a novel ranking approach called SHKS, which builds upon the strengths of the k-shell decomposition method and incorporates the concept of structural holes (SH) [30]. Although the concept of structural holes can improve influence assessment, its computational complexity is relatively high, and it may not effectively handle networks with dense nodes or complex relationships. Liang et al. (2024) proposed a novel measurement of node centrality based on degree, quadrilateral-containing clustering coefficient and k-shell decomposition value(DCK) [31]. This method has not been widely validated in high-dimensional complex networks and may suffer from overfitting of local structures.

Although a large number of node ranking methods based on k-shell have been proposed so far, finding a widely accepted approach that strikes a balance between ranking accuracy and efficiency is still an ongoing exploration. Considering the factors mentioned above, this paper proposes a new method KTD that combines the node’s iteration factor, global k-shell value, local degree value, and neighbor information, overcoming the limitations of existing methods that cannot dynamically adjust and have a single focus on local topological information. We summarize the core contributions of this research as follows: (i) The KTD algorithm uses iterative information from the K-shell decomposition process, along with local node degree, to refine the hierarchy of nodes with the same K-shell value. During the initial influence assessment, both global network position and local topological information are fully utilized. This dynamic capture of the node’s topological evolution allows the algorithm to flexibly adapt to changes in network structure, providing a more accurate reflection of the node’s propagation capability in complex networks. (ii) In the final influence calculation, both global and local information from all neighboring nodes are integrated. The algorithm comprehensively considers factors such as the current node’s degree, the degree of its neighbors, and the iteration factor, thereby enhancing the influence of local information on global propagation. This approach effectively identifies key nodes that are often overlooked in large-scale complex networks, particularly in networks with strong local or community structures, providing a more accurate assessment of node influence. (iii) Simulation experiments on eight real-world networks demonstrate that the KTD algorithm outperforms other baseline methods in terms of both monotonicity and accuracy, while maintaining low time complexity.

The rest of this paper is organized as follows: The proposed KTD algorithm is presented in the Method section. The Experimental analysis section discusses the experimental results of the proposed KTD method. Finally, The Conclusions section provides the conclusion and some future recommendations of our study.

## Method

The algorithm proposed by Wang et al. [24] performs K-shell decomposition on a network by calculating node influence based on both the node’s iteration factor and K-shell value. The specific steps are as follows:

Delete all nodes with a degree of 1 and their corresponding edges in the network. These nodes are assigned an iteration factor (IT) of 1. Next, Continue by deleting all remaining nodes with a degree of 1 and their edges. These nodes are assigned an iteration factor (IT) of 2. This process continues until there are no nodes with degree 1 in the network. During this process, the iteration factor IT increases in sequence, and all deleted nodes have a K-shell (KS) value of 1. Following the same method, delete nodes with degrees 2, 3, 4, etc. until all nodes are deleted.

**Fig 1:**
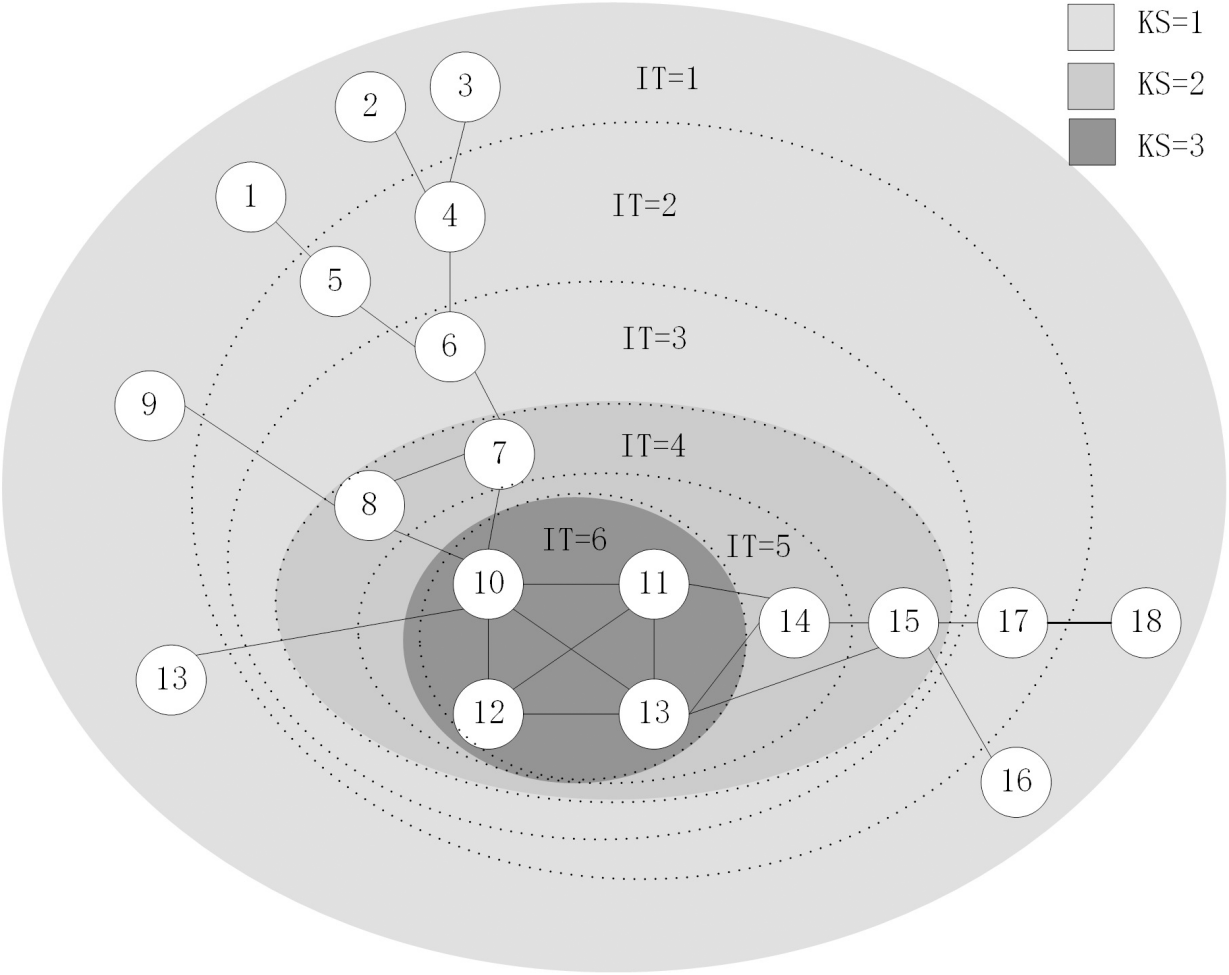
K-shell decomposition and iteration results for an example network.

The example network has 18 nodes and 24 edges. It is decomposed by k-shell into three K-shell layers and six iteration layers (Fig 1).

Table 1 shows the iteration factor (IT), K-shell value (KS), and degree value for all nodes in the example network. It can be observed that nodes 4 and 5 have the same IT and KS values. If only these global position factors were considered, both nodes would have the same influence. However, when local factors such as node degree are taken into account, node 4, having a higher degree, has a greater influence compared to node 5, which is more realistic. Similarly, nodes 10, 11, 12, and 13 have the same IT and KS values, but their degree values differ. Node 10 has the highest degree, while node 12 has the lowest. Therefore, among these four nodes, node 10 has the highest influence, and node 12 has the lowest. For nodes 7 and 8, the IT, KS values, and degree values are identical. However, considering the neighbors’ information reveals that node 7’s neighbor, node 6, has an IT of 3 and a degree of 3, while node 8’s neighbor, node 9, has an IT of 1 and a degree of 1. After considering the neighbor information, it is clear that node 7 has higher influence than node 8.

**Table 1:** Iteration Factor (IT), K-shell Value (KS), and degree value for nodes in the example network.

Node Number	IT	KS	d
1	1	1	1
2	1	1	1
3	1	1	1
4	2	1	3
5	2	1	2
6	3	1	3
7	4	2	3
8	4	2	3
9	1	1	1
10	6	3	6
11	6	3	4
12	6	3	3
13	6	3	5
14	5	2	3
15	4	2	4
16	1	1	1
17	2	1	2
18	1	1	1

Therefore, unlike the KS_IF method, which incorporates the degree value in the final influence calculation, we attempt to include the impact of degree in the initial influence calculation, using both the iteration factor and degree to refine the distinction between nodes with the same K-shell value. This allows us to account for the degree influence of the current node as well as all its neighboring nodes, enhancing the local topological factors in complex networks. As a result, we propose a novel method, KTD, which combines global topology (K-shell value) with local topology (degree value and neighbor information), while also considering the iteration factor of each node. The algorithm is outlined as follows:

(i) **K-shell Decomposition and Iteration Factor Calculation**: First, the network is decomposed using K-shell decomposition, and the K-shell value for each node is computed. Simultaneously, following the method proposed by Hébert-Dufresne et al. [32], the network undergoes iterative processing. The iteration factor (IT) for each node increases with each decomposition step, reflecting the dynamic change in the node’s position within the network.
(ii) **Preliminary Node Influence Assessment**: Using the K-shell value and the iteration factor, the direct influence of each node is calculated. The influence of a node is not solely dependent on its K-shell value; it is also influenced by its position within the iterative process. Furthermore, the node’s degree value is used to further adjust the influence calculation, accounting for both global and local factors in determining the node’s importance.

**Definition 1:** Given a complex network *G*, each node is assigned a K-shell Value (KS) by the K-shell decomposition. The maximum iteration factor in the network is denoted as ${IT}_{Max}$, and the maximum degree value is denoted as $\deg_{Max}$. Suppose node $v_{i}\in G$, the ks value of $v_{i}$ is ${KS}_{i}$, the iteration factor (IT) is ${IT}_{i}$, and the degree value is $\deg_{i}$. The direct influence of node $v_{i}$, denoted as $C_{v_{i}}$, is defined as follows:


$$C_{v_{i}}={KS}_{i}+\frac{{IT}_{i}}{{IT}_{Max}}*\frac{\deg_{i}}{\deg_{Max}}$$ (1)

In this calculation, the iteration factor and degree are used to refine the distinction between nodes with the same K-shell value. The product of the iteration factor and degree emphasizes the combined impact of the node’s global position and its local connectivity. The iteration factor reflects the importance of the node in the global network structure, while the degree reflects its local connectivity. By multiplying these two factors and normalizing them using the maximum iteration factor and degree values, the influence value is ensured to have an appropriate scale, resulting in a more balanced and reasonable measure of the node’s direct influence.

(iii ) **Final Influence Calculation:** The KTD method further refines node influence by considering neighbor information. The influence of neighbor nodes, along with their iteration factors and degree values, affects the final influence of the target node, enabling a more accurate assessment of the node’s relative influence within the network.

**Definition 2:** Given a complex network *G*, Suppose node $v_{j}\in N_{i}$, $N_{i}$ is the set of neighbor nodes of node $v_{i}$. The final influence of node $v_{i}$, denoted as $E_{v_{i}}$, is defined as follows:


$$\begin{array}{c} E_{v_{i}}\;=\;C_{v_{i}}\;+\;\sum {\mathrm{\backslash nolimits}}_{v_{j}\in N_{i}}C_{v_{j}}\; \end{array}$$ (2)

## Experimental analysis

In this section, experiments and simulations are conducted on eight commonly used networks to compare and analyze the performance of the KTD algorithm with other ranking algorithms from three aspects: monotonicity, effectiveness, and time performance.

### Datasets

The eight commonly used networks are: (1) Karate network [33], the Zachary Karate club network; (2) Dolphins network [34], the social network of bottlenose dolphins; (3) Jazz network [35], jazz musicians network; (4) USAir97 network [36], the air route network in the United States in 1997; (5) Euroroad network [37], the international E-road network; (6) Hamster network [38], friendships and family links between users of the website hamsterster.com; (7) power-US-Grid [39], information about the power grid in the western states of the USA; (8) Hepth [40], collaboration network in high-energy physics. The statistical properties of the network datasets are shown in Table 2, where *N* represents the number of nodes in the network, *E* denotes the number of edges, *k* indicates the average degree, *d* represents the average shortest distance, *c* denotes the clustering coefficient, ${Ks}_{\max}$ indicates the maximum core value from K-shell decomposition, and $\beta_{th}$ represents the popularity threshold of the network.

**Table 2:** Statistical characteristics of eight complex networks.

Network	*N*	*E*	*k*	*d*	*c*	${Ks}_{\max}$	$\beta_{th}$
karate	34	78	4.59	2.41	0.57	4	0.129
dolphins	62	159	5.13	3.36	0.26	4	0.147
jazz	198	2 472	27.7	2.24	0.62	29	0.026
USair	332	2 126	12.81	2.74	0.63	26	0.022
Euroroad	1174	1417	2.41	2.42	0.17	2	0.333
hamster	2426	16 631	13.71	3.67	0.54	24	0.024
power-US-Grid	4941	6594	5.13	18.99	0.08	5	0.26
Hepth	8361	15751	3.77	7.03	0.64	24	0.115

### Monotonicity

The monotonicity index *M(R)*[41] is used to evaluate the algorithm’s ability to distinguish. Higher monotonicity indicates stronger discriminative ability. *M(R)* is calculated using the following formula:


$$\;M(R)\;=\left(1-\frac{\sum_{r\in R}n_{r}(n_{r}-1)}{n(n\;-\;1)}\right)2$$ (3)

In Formula (3), *R* represents the data vector to be ranked, n denotes the number of distinct ranks in *R*, and $n_{r}$ represents the number of nodes with the same rank. If all nodes have their influence values assigned to the same rank, then *M = 0*, indicating the lowest level of discriminative power. Conversely, if all nodes have their influence values assigned to different ranks, then *M = 1*, indicating the highest level of discriminative power.

Table 3 shows the monotonicity of nine ranking algorithms—dc, bc, cc, k-shell, Ks + , KSIF, LGI, DCK, and KTD—in eight networks. It is evident that the KTD algorithm has the highest monotonicity metric *M* in six networks: karate, dolphins, jazz, USair, hamster and Hepth. Notably, in the dolphins, jazz, USAir and Hepth networks, *M* is close to 1. This indicates that the KTD algorithm provides a high level of discriminative power for node influence ranking.

**Table 3:** The monotonicity performance applied to eight complex networks.

Network	M(dc)	M(bc)	M(cc)	M(ks)	M(ks+)	M(KSIF)	M(LGI)	M(DCK)	M(KTD)
karate	0.7079	0.7754	0.8993	0.4958	0.7413	**0.9542**	0.9412	0.9541	**0.9542**
dolphins	0.8312	0.9623	0.9737	0.3769	0.8564	**0.9968**	0.9958	0.9958	**0.9968**
jazz	0.9659	0.9885	0.9878	0.7944	0.9880	0.9993	0.9994	0.9994	**0.9995**
USair	0.8586	0.6970	0.9892	0.8114	0.8861	0.9946	0.9940	0.9940	**0.9948**
Euroroad	0.4442	0.9374	**0.9988**	0.2129	0.6311	0.9715	0.9513	0.9512	0.9714
hamster	0.8980	0.7123	0.9851	0.8714	0.9268	**0.9857**	**0.9857**	**0.9857**	**0.9857**
power-US-Grid	0.5924	0.8319	**0.9998**	0.2459	0.69287	0.6600	0.9801	0.9895	0.9842
Hepth	0.5926	0.8313	0.9918	0.2459	0.6600	0.9837	0.9801	0.9895	**0.9941**

**Fig 2:**
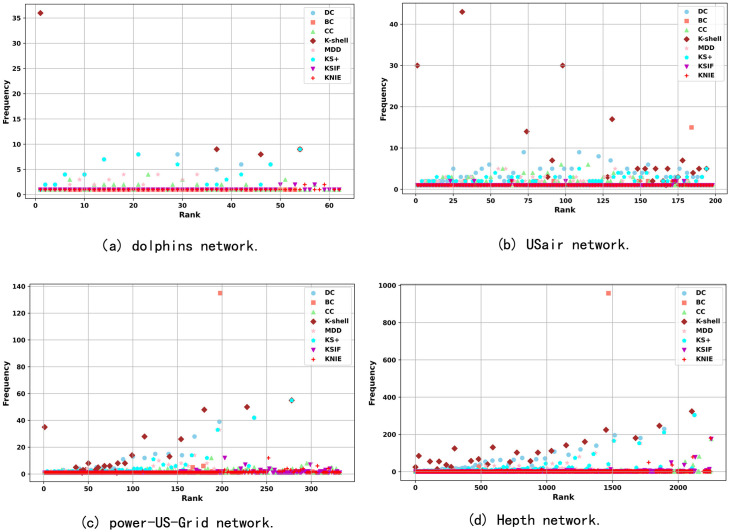
The frequency distribution of ranking levels for different algorithms.

Additionally, to more intuitively demonstrate the discriminative power of different algorithms for node influence, we analyzed the number of node ranks and the frequency distribution of the number of nodes in each rank on four networks (Fig 2). The KTD algorithm results in a higher number of ranks on the four networks, with each rank covering fewer nodes compared to other methods. This indicates that the KTD algorithm can better differentiate node influence.

**Fig 3:**
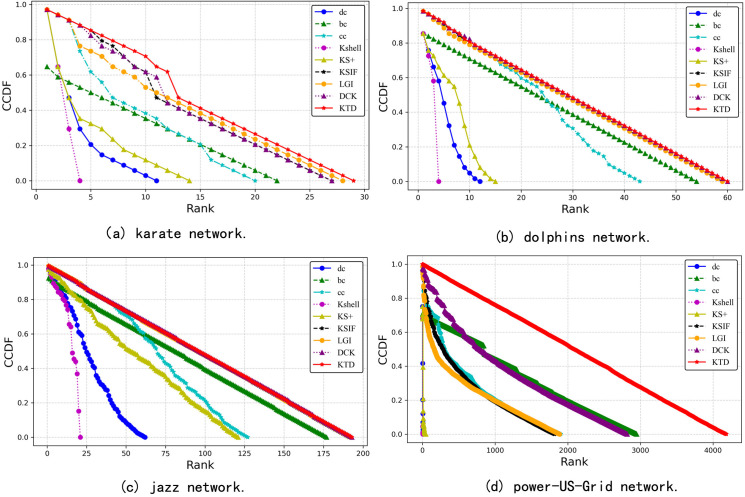
The CCDF curves of different algorithms.

To better assess the discriminative power of the algorithm, we employed the complementary cumulative distribution function (CCDF) to plot the distribution curve on four networks (Fig 3). When many nodes exhibit similar rankings, the CCDF curve decreases rapidly. Conversely, if the rankings are more evenly distributed, the CCDF curve declines gradually. In the Karate and Power-US-Grid networks, the KTD algorithm performs the best, as indicated by its CCDF curve gradually decreasing along the diagonal, suggesting a wide and balanced distribution of influence scores. In the Dolphins and Jazz networks, LGI, DCK, KSIF, and KTD all exhibit strong performance, with their CCDF curves nearly overlapping. Overall, the KTD algorithm demonstrates robust performance across all four networks and can effectively distinguish the influence of nodes.

### Effectiveness

To evaluate the effectiveness of the algorithms, the ranking results obtained from the seven ranking algorithms—dc, bc, cc, k-shell, Ks + , KSIF, LGI, DCK and KTD—on the eight networks are compared with the rankings simulated using the SIR model [42]. The Kendall rank correlation coefficient [43] is calculated for this comparison.

The SIR model is a classic infectious disease model that categorizes network nodes into three states:

**Susceptible (S)**: Nodes in this state have a certain probability of being infected by neighboring nodes.

**Infected (I)**: Nodes in this state are infected and can spread the infection to others.

**Recovered (R)**: Nodes in this state have recovered from the infection and no longer have the ability to spread it.

At the start of the spread, nodes in the infected state (I) infect neighboring nodes in the susceptible state (S) with a probability *β*. Nodes in the infected state (I) transition to the recovered state (R) with a probability *γ*, achieving immunity. This process is repeated until the network reaches a stable state. At the end of the SIR process, the number of nodes in the recovered state is considered as the node’s propagation capability. By repeating this process, the propagation capability of each node is calculated, and the SIR ranking list is obtained by sorting the nodes in descending order.

When the infection probability *β* is high, the disease can spread extensively across the network. When *β* is low, the disease only spreads within a small area [44]. For the disease to spread and become endemic in the network, the infection probability must be greater than the epidemic threshold. The epidemic threshold $\beta_{th}$ is defined as:


$$\beta_{th}\;\approx \;\frac{k}{k^{2}}\;$$ (4)

where *k* is the average degree of the network, and $k^{2}$ is the average second-order degree of the network.

The Kendall rank correlation coefficient τ is used to measure the correlation between two ranking sequences, with values ranging from −1–1. A higher *τ* indicates a stronger correlation between the two ranking sequences. The formula for calculating *τ* is as follows:


$$\begin{array}{c} \;\tau (X,Y)=\frac{2(N_{c}-N_{d})}{n(n-1)} \end{array}$$ (5)

where *X* and *Y* are the two ranking sequences, $N_{c}$ represents the number of concordant pairs (i.e., pairs where the order is the same in both sequences), $N_{d}$ represents the number of discordant pairs (i.e., pairs where the order is different), and *n* is the number of nodes in the network.

In this experiment, a single node was used as the source of infection on eight networks, with the infection probability *β* slightly greater than the epidemic threshold $\beta_{th}$, to facilitate SIR propagation. When the network reached a stable state, the number of nodes in the recovered state R was used to reflect the influence of the initial node. To more accurately calculate node influence, all network nodes underwent 100 independent repeated experiments and the average value was calculated.

We calculated the Kendall correlation coefficients between the ranking results of different algorithms and the rankings obtained from the SIR model, where *β* is the infection probability of the network (Table 4). In networks such as karate (*τ* = 0.8645), dolphins (*τ* = 0.8360), jazz (*τ* = 0.8687), and USair (*τ* = 0.9176), the KTD algorithm outperforms other methods in terms of Kendall coefficient values, with an average improvement of 5.15% over the second-best method. This indicates that in these networks, the node influence ranking produced by KTD aligns more closely with the actual diffusion in the SIR model, showing higher accuracy. However, in the Euroroad network, KTD performs second only to DCK, and in the power-US-Grid network, KTD performs second only to KSIF. This suggests that, although KTD outperforms traditional algorithms in all networks, it may perform worse than certain specific algorithms in networks with uneven node distribution, irregular topologies, or higher-order structures (such as triangles and quadrilaterals). Overall, KTD achieves the highest Kendall coefficient values in most networks, indicating it has higher accuracy in identifying influential nodes compared to other methods.

**Table 4:** Kendall coefficient values for the ranking results of different algorithms compared to the SIR model rankings.

Network	*β*	*τ*(dc)	*τ*(bc)	*τ*(cc)	*τ*(ks)	*τ*(ks+)	*τ*(KSIF)	*τ*(LGI)	*τ*(DCK)	*τ*(KTD)
karate	0.15	0.7504	0.6435	0.8004	0.6150	0.7647	0.6435	0.5829	0.5401	**0.8645**
dolphins	0.15	0.7811	0.5917	0.6033	0.488	0.7884	0.7884	0.7493	0.7852	**0.8360**
jazz	0.03	0.7918	0.5311	0.7219	0.6834	0.7889	0.7677	0.7681	0.8153	**0.8687**
USair	0.03	0.7178	0.4954	0.7913	0.7283	0.7456	0.8560	0.8441	0.8580	**0.9176**
Euroroad	0.35	0.4740	0.3168	0.6599	0.4903	0.5333	0.6512	0.6648	**0.6743**	0.6653
hamster	0.03	0.7029	0.5012	0.7688	0.6919	0.7011	0.8339	0.8082	0.8365	**0.8467**
power-US-Grid	0.3	0.4439	0.3014	0.3344	0.3428	0.4658	**0.6327**	0.4463	0.4892	0.6126
Hepth	0.12	0.5635	0.3466	0.7593	0.5718	0.5834	0.7502	0.7508	0.7415	**0.7789**

The proposed index better expresses the diffusion power of the SIR model because it simultaneously considers both global and local network structures. Unlike the KS_IF method, which refines nodes with the same K-shell value using the iteration factor, our method incorporates both the iteration factor and node degree. Although the KS_IF method also considers the degree of the current node when calculating the final influence, we place the degree in the initial step of the preliminary influence evaluation. This allows the final influence to take into account both the current node’s degree and the degree of all its neighboring nodes. This combined approach enables our index to capture node influence more comprehensively, leading to better alignment with the SIR propagation experiment.

**Fig 4:**
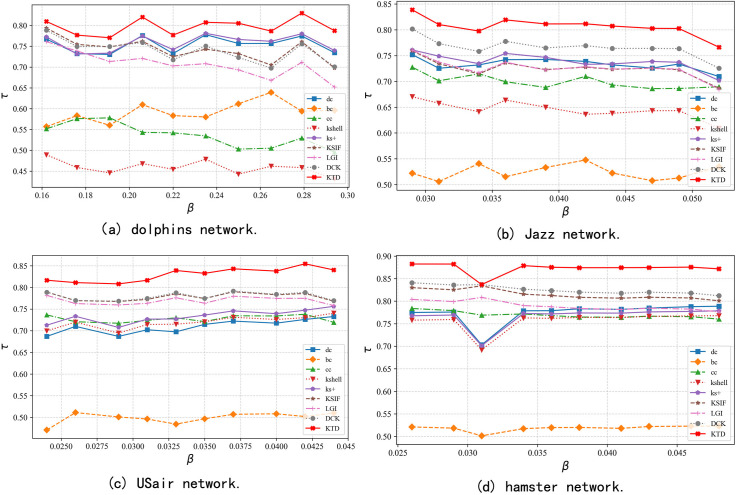
The variation curves of Kendall coefficient between the ranking results of different methods and the SIR rankings under different infection rates.

In the following experiments, by varying the infection rate *β* from $\beta_{th}$ to 2×$\beta_{th}$ in increments of 0.1×$\beta_{th}$, SIR simulations are conducted on the dolphins, jazz, USair, and Euroroad networks. The changes in Kendall coefficient between the ranking results of different methods and the SIR rankings under different infection rates are presented in Fig 4. The KTD algorithm clearly demonstrates the best performance across all four networks under different infection rates, highlighting its superior effectiveness in accurately ranking node influence compared to other algorithms.

**Fig 5:**
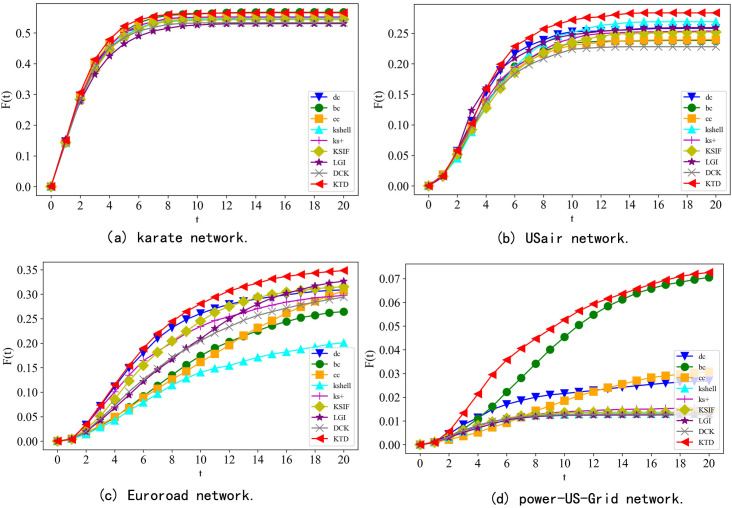
The SIR propagation curves for the Top-10 influential nodes identified.

In many practical applications, besides ranking all nodes by their influence, greater emphasis is placed on identifying key nodes. In four networks, the Top-10 nodes with the highest influence according to various algorithms are selected as infection sources. With an infection probability *β* slightly greater than the epidemic threshold $\beta_{th}$ and an immunity rate *γ* set to 0.5, SIR propagation is conducted. The cumulative number of immune nodes is plotted as a function of time, based on 100 independent repetitions, and the average is calculated to produce the SIR transmission curve (Fig 5).

It can be observed that in the karate network, the SIR propagation curve of the KTD algorithm is slightly lower than that of the BC algorithm. The propagation capability of the top-10 nodes is second only to the BC algorithm and higher than that of other algorithms. As the number of network nodes increases, in the USair, Euroroad, and power-US-Grid networks, the SIR propagation curve of the KTD algorithm is higher than that of other algorithms, indicating that the propagation capability of the top-10 nodes is superior to that of other algorithms. The experimental results show that the KTD algorithm has a high accuracy in identifying key nodes, especially in complex networks.

### Time performance

To evaluate the time performance of the KTD algorithm, we compared the execution times of different algorithms in eight networks. The experimental setup consisted of a computer with an 11th Gen Intel® Core™ i7-1160G7 @ 1.20GHz processor, 16GB RAM, 64-bit Windows 11, and Python version 3.8.

Table 5 presents the average execution times, based on 100 independent repetitions, for different algorithms in the eight networks. In all eight networks, the dc, k-shell, and ks+ algorithms have the shortest execution times; however, their accuracy and discriminatory power are insufficient. The runtime of the DCK algorithm does not exhibit a direct correlation with the number of nodes or edges, as it depends on the number of triangles and quadrilaterals in the network. Therefore, it is not included in the comparison of runtimes. Besides these, KSIF and KTD algorithms show the best time performance, with execution times less than those of the bc, cc, and LGI algorithms. For networks with fewer than 100 nodes, such as karate and dolphins, the KTD algorithm’s execution time is equivalent to that of KSIF. However, as the number of nodes increases, the KTD algorithm performs faster than KSIF in the other four networks. The results indicate that while the KTD algorithm’s time performance is inferior to dc, k-shell, and ks + , it is superior to other algorithms. Overall, the KTD algorithm demonstrates good time efficiency.

**Table 5:** The execution time T of different algorithms on eight networks.

Network	T(dc)	T(bc)	T(cc)	T(ks)	T(ks+)	T(KSIF)	T(LGI)	T(DCK)	T(KTD)
karate	9.98e-06	0.0024	0.0007	0.0001	0.0001	0.0005	0.0816	0.0009	0.0005
dolphins	1.99e-05	0.0085	0.0022	0.0002	0.0002	0.0013	0.2975	0.0020	0.0013
jazz	5.03e-05	0.2029	0.0550	0.0026	0.0025	0.0185	189.23	0.4276	0.0168
USair	6.19e-05	0.3582	0.0877	0.0024	0.0028	0.0208	143.78	0.5695	0.0192
Euroroad	0.0002	2.0127	0.4384	0.0025	0.0038	0.1304	33.521	0.0156	0.1218
hamster	0.0005	17.499	4.2744	0.0141	0.0173	0.6977	3249.4	5.1875	0.6396
power-US-Grid	0.0010	65.973	24.931	0.0314	0.0298	4.1732	792.68	0.0802	4.0331
Hepth	0.0019	168.52	32.080	0.0337	0.0468	8.8042	3656.6	0.3754	7.7555

## Conclusions

Accurately identifying key nodes in complex networks holds significant research value and has broad applications in various fields such as social networks, biology, and information dissemination. This paper proposes a novel node influence ranking algorithm that integrates iterative K-shell decomposition, node degree, and neighbor information. The method fully leverages the efficiency of K-shell decomposition and the advantages of global positioning through iteration factors, while highlighting local topological details by incorporating node degree and neighbor information, thus improving the accuracy of node influence ranking. Experimental results show that, compared to other methods, the proposed algorithm performs better in terms of accuracy and monotonicity. It proves effective in identifying key nodes while maintaining reasonable time complexity.

However, this study has some limitations, such as the lack of error analysis and sensitivity testing for the algorithm’s performance. Currently, the algorithm’s performance primarily relies on the K-shell value, node degree, and iteration factor of the network. However, the specific impact of variations in these factors on the algorithm’s performance has not been thoroughly investigated. Therefore, future research should conduct comprehensive error analysis and sensitivity testing to explore the effects of these key factors on the algorithm’s performance, aiming to further optimize its robustness and adaptability. Additionally, it is necessary to validate the KTD algorithm on larger and more diverse datasets across different domains, which will help improve its effectiveness and generalizability. Furthermore, We will also continue to explore potential alternative calculation methods that combine the K-shell value, node degree, and iteration factor in future research, with the aim of developing a more optimal node influence ranking method.

## Supporting information

S1 Table The ranking results of the seven algorithms on the karate network. (XLSX)

S2 Table The ranking results of the seven algorithms on the dolphins network. (XLSX)

S3 Table The ranking results of the seven algorithms on the jazz network. (XLSX)

S4 Table The ranking results of the seven algorithms on the USair network. (XLSX)

S5 Table The ranking results of the seven algorithms on the Euroroad network. (XLSX)

S6 Table The ranking results of the seven algorithms on the hamster network. (XLSX)

S7 Table The ranking results of the seven algorithms on the power-US-Grid network. (XLSX)

S8 Table The ranking results of the seven algorithms on the Hepth network. (XLSX)

## References

[1] Chen R, Wu Q, Guo W, Guo K, Wang Q. Overlapping Community Discovery Based on the Combination of Node Influence and β-Connected Neighbors. International Journal of Cooperative Information Systems. 2019;28(4):1950011.

[2] Ren X, L LL. Review of ranking nodes in complex networks. Chinese Science Bulletin. 2014;59(13):1175–97.

[3] Fang X, Misra S, Xue G, Yang D. Smart Grid — The New and Improved Power Grid: A Survey. IEEE Commun Surv Tutorials. 2012;14(4):944–80. doi: 10.1109/surv.2011.101911.00087

[4] Pagani GA, Aiello M. The Power Gridas a complex network: A survey. Physica A: Statistical Mechanics and its Applications. 2013;392(11):2688–700.

[5] Ni Q, Guo J, Huang C, Wu W. Community-based rumor blocking maximization in social networks: Algorithms and analysis. Theoretical Computer Science. 2020;840(10):257–69. 32939100 10.1016/j.tcs.2020.08.030 PMC7482597

[6] Hu SYNFJ. mode1ing the spread of infectious diseases through inf1uence maximization. Optimization Letters. 2022;16(5):1563–86. 35573937 10.1007/s11590-022-01853-1 PMC9091155

[7] Albert R, Barabasi AL. Statistical mechanics of complex networks. Reviews of Modern Physics. 2002;26(1):xii.

[8] Freeman LC. Centrality in social networks conceptual clarification. Social Networks. 1978;1(3):215–39. doi: 10.1016/0378-8733(78)90021-7

[9] Chen D, Lü L, Shang M-S, Zhang Y-C, Zhou T. Identifying influential nodes in complex networks. Physica A: Statistical Mechanics and Its Applications. 2012;391(4):1777–87.

[10] Lü L, Zhou T, Zhang Q-M, Stanley HE. The H-index of a network node and its relation to degree and coreness. Nature communications. 2016;7(1):10168. 10.1038/ncomms10168 PMC4729922 26754161

[11] Berahmand K, Bouyer A, Samadi N. A new local and multidimensional ranking measure to detect spreaders in social networks. Computing. 2019;101(11):1711–33.

[12] Freeman LC. A set of measures of centrality based on betweenness. Sociometry. 1977;40(1):35–41.

[13] Sabidussi G. The centrality index of a graph. Psychometrika. 1966;31(4):581–603. 5232444 10.1007/BF02289527

[14] Mijia Li, Hongquan Wei, Yingle Li, Shuxin Liu. Key Node identification method in Social Networks Based on Improved K-Shell. Computer Applications and Software. 2023;40(7):305–10.

[15] Bonacich P. Technique for analyzing overlapping memberships. Sociological methodology. 1972;4:176–85.

[16] Brin S, Page L. The anatomy of a large-scale hypertextual Web search engine. Computer Networks and ISDN Systems. 1998;30(1–7):107–17. doi: 10.1016/s0169-7552(98)00110-x

[17] Lü L, Zhang YC, Yeung CH, Zhou T. Leaders in social networks, the delicious case. PloS One. 2011;6(6): 1–9. 10.1371/journal.pone.0021202 PMC3124485 21738620

[18] Seshu Chakravarthy T, Selvaraj L. HIKS: A K‐shell‐weighted hybrid approach method for detecting influential nodes in complex networks using possible edge weights. Int J Communication. 2024;37(7). doi: 10.1002/dac.5722

[19] Kitsak M, Gallos LK, Havlin S, Liljeros F, Muchnik L, Stanley HE, et al. Identification of influential spreaders in complex networks. Nature Phys. 2010;6(11):888–93. doi: 10.1038/nphys1746

[20] Wang T, Liang Z, Zhang R. A complex network node importance evaluation method based on information entropy and iterative factors. Acta Physica Sinica. 2023;72(4):048901.

[21] Zeng A, Zhang CJ. Ranking spreaders by decomposing complex networks. Physics Letters A. 2013;377(14):1031–5.

[22] Bae J, Kim S. Identifying and ranking influential spreaders in complex networks by neighborhood coreness. Physica A: Statistical Mechanics and its Applications. 2014;395:549–59.

[23] Qiu L, Zhang J, Tian X. Ranking influential nodes in complex networks based on local and global structures. Applied Intelligence. 2021;51:4394–407.

[24] Wang Z, Zhao Y, Xi J, Du C. Fast ranking influential nodes in complex networks using a k-shell iteration factor. Physica A: Statistical Mechanics and its Applications. 2016;461:171–81. doi: 10.1016/j.physa.2016.05.048

[25] Zareie A h m a d, Sheikhahmadi, et al. Finding influential nodes in social networks based on neighborhood correlation coefficient. Knowledge-Based Systems. 2020;194:105580.

[26] Wang TT, Liang ZW, Zhang RX. Importance evaluation method of complex network nodes Based on information entropy and iteration factor. Acta Physica Sinica. 2023;72(4):331–41.

[27] Zareie A, Sheikhahmadi A. A hierarchical approach for influential node ranking in complex social networks. Expert Systems with Applications. 2018;93:200–11. doi: 10.1016/j.eswa.2017.10.018

[28] Ullah A, Wang B, Sheng JF, Long J, Sun ZJ. Identifying vital nodes from local and global perspectives in complex networks. Expert Systems with Applications. 2021;186(1):115778.

[29] HamaKarim BR, Mohammadiani RP, Sheikhahmadi A, Hamakarim BR, Bahrami M. A method based on k-shell decomposition to identify influential nodes in complex networks. J Supercomput. 2023;79(14):15597–622. doi: 10.1007/s11227-023-05296-y

[30] Zhao Z, Li D, Sun Y, Zhang R, Liu J. Ranking influential spreaders based on both node k-shell and structural hole. Knowl Based Syst. 2023;260:110163.

[31] Liang L, Tang Z, Gong S. Identifying influential spreaders in complex networks based on local and global structure. Journal of Computational Science. 2024;82:102395. doi: 10.1016/j.jocs.2024.102395

[32] Hébert-Dufresne L, Grochow JA, Allard A. Multi-scale structure and topological anomaly detection via a new network statistic: The onion decomposition. Scientific Reports. 2016;6(1): 31708. 27535466 10.1038/srep31708 PMC4989159

[33] Zachary WW. An information flow model for conflict and fission in small groups. Journal of Anthropological Research. 1977;33(4):452–73.

[34] Lusseau D, Schneider K, Boisseau OJ, Haase P, Slooten E, Dawson SM. The bottlenose dolphin community of Doubtful Sound features a large proportion of long-lasting associations. Behavioral Ecology and Sociobiology. 2003;54(4):396–405. doi: 10.1007/s00265-003-0651-y

[35] Gleiser PM, Danon L. Community structure in jazz. Advances in Complex Systems. 2003;6(04):565–73.

[36] Colizza V, Pastor-Satorras R, Vespignani A. Reaction–diffusion processes and metapopulation models in heterogeneous networks. Nature Physics. 2007;3(4):276–82.

[37] Eash R, Chon K, Lee Y, Boyce D. Equilibrium traffic assignment on an aggregated highway network for sketch planning. Transportation Research. 1979;13:243–57.

[38] Kunegis J. Konect: the koblenz network collection. In: Proceedings of the 22nd international conference on world wide web, 2013.

[39] Newman M. Network data. http://www-personal.umich.edu/mejn/netdata/. 2022.

[40] Newman MEJ. The structure of scientific collaboration networks. Proceedings of the National Academy of Sciences of the United States of America. 2000;98(2):404–9. 10.1073/pnas.021544898 PMC14598 11149952

[41] Maji G, Mandal S, Sen S. A systematic survey on influential spreaders identification in complex networks with a focus on K-shell based techniques. Expert Systems with Applications. 2020;161:113681. doi: 10.1016/j.eswa.2020.113681

[42] Ibnoulouafi A, El Haziti M, Cherifi H. M-Centrality: identifying key nodes based on global position and local degree variation. J Stat Mech. 2018;2018(7):073407. doi: 10.1088/1742-5468/aace08

[43] Maji G. Influential spreaders identification in complex networks with potential edge weight based k-shell degree neighborhood method. Journal of Computational Science. 2020;39:101055.

[44] Zareie A, Sheikhahmadi A, Fatemi A. Influential nodes ranking in complex networks: An entropy-based approach. Chaos, Solitons & Fractals. 2017;104:485–94.

